# Transcatheter aortic valve implantation in patients with significant septal hypertrophy

**DOI:** 10.1007/s00392-024-02432-3

**Published:** 2024-03-11

**Authors:** Martin Beyer, Till Joscha Demal, Oliver D. Bhadra, Matthias Linder, Sebastian Ludwig, David Grundmann, Lisa Voigtlaender-Buschmann, Lara Waldschmidt, Johannes Schirmer, Niklas Schofer, Simon Pecha, Stefan Blankenberg, Hermann Reichenspurner, Lenard Conradi, Moritz Seiffert, Andreas Schaefer

**Affiliations:** 1 Department of Cardiovascular Surgery, University Heart & Vascular Center Hamburg, Martinistraße 52, 20246 Hamburg, Germany; 2 Department of Cardiology, University Heart & Vascular Center Hamburg, 20246 Hamburg, Germany

**Keywords:** TAVR, TAVI, Septal hypertrophy, Aortic valve stenosis

## Abstract

**Background:**

Previous reports suggest septal hypertrophy with an interventricular septum depth (IVSD) ≥ 14 mm may adversely affect outcomes after transcatheter aortic valve implantation (TAVI) due to suboptimal valve placement, valve migration, or residual increased LVOT pressure gradients.

**Aims:**

This analysis investigates the impact of interventricular septal hypertrophy on acute outcomes after TAVI.

**Methods:**

Between 2009 and 2021, 1033 consecutive patients (55.8% male, 80.5 ± 6.7 years, EuroSCORE II 6.3 ± 6.5%) with documented IVSD underwent TAVI at our center and were included for analysis. Baseline, periprocedural, and 30-day outcome parameters of patients with normal IVSD (< 14 mm; group 1) and increased IVSD (≥ 14 mm; group 2) were compared. Data were retrospectively analyzed according to updated Valve Academic Research Consortium-3 (VARC-3) definitions. Comparison of outcome parameters was adjusted for baseline differences between groups using logistic and linear regression analyses.

**Results:**

Of 1033 patients, 585 and 448 patients were allocated to groups 1 and 2, respectively. There was no significant difference between groups regarding transfemoral access rate (82.6% (*n* = 478) vs. 86.0% (*n* = 381), *p* = 0.157). Postprocedural mean transvalvular pressure gradient was significantly increased in group 2 (group 1, 7.8 ± 4.1 mmHg, vs. group 2, 8.9 ± 4.9 mmHg, *p* = 0.046). Despite this finding, there was no significant difference between groups regarding the rates of VARC-3 adjudicated composite endpoint device success (90.0% (*n* = 522) vs. 87.6% (*n* = 388), *p* = 0.538) or technical success (92.6% (*n* = 542) vs. 92.6% (*n* = 415), *p* = 0.639). Moreover, the groups showed no significant differences regarding the rates of paravalvular leakage ≥ moderate (3.1% (*n* = 14) vs. 2.6% (*n* = 9), *p* = 0.993), postprocedural permanent pacemaker implantation (13.4% (*n* = 77) vs. 13.8% (*n* = 61), *p* = 0.778), or 30-day mortality (5.1% (*n* = 30) vs. 4.5% (*n* = 20), *p* = 0.758).

**Conclusion:**

Although transvalvular mean pressure gradients were significantly higher in patients with increased IVSD after TAVI, acute outcomes were comparable between groups suggesting no early impact of adverse hemodynamics due to elevated IVSD. However, how these differences in hemodynamic findings may affect mid- and long-term outcomes, especially in terms of valve durability, needs to be evaluated in further investigations.

**Graphical Abstract:**

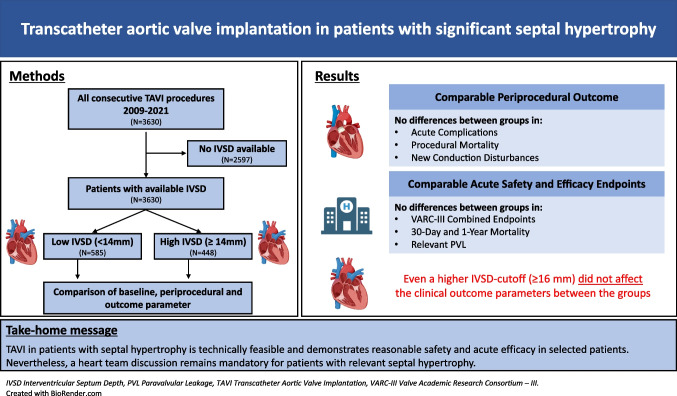

**Supplementary Information:**

The online version contains supplementary material available at 10.1007/s00392-024-02432-3.

## Introduction

Transcatheter aortic valve implantation (TAVI) is a well-established therapy for severe aortic stenosis (AS) across all risk strata according to current guidelines for the management of valvular heart disease [1, 2]. Multiple clinical risk factors for poor outcomes after TAVI like age, frailty, sarcopenia, malnutrition, or chronic kidney disease have been described in the past [3–6]. However, identification of patient-specific anatomical characteristics and subsequent adaption of these circumstances in procedural planning and prosthesis selection may be even more important in terms of acute procedural and technical success in TAVI with regard to the incidence of paravalvular leakage (PVL), conduction disturbances with need for permanent pacemaker implantation (PPM), or transcatheter heart valve (THV) dysfunction [7–10].

Previous studies including the Framingham Heart Study have defined septal hypertrophy as an interventricular septum depth (IVSD) of ≥ 14 mm, which can be caused by different conditions such as long-standing arterial hypertension, AS, or genetic disorders like hypertrophic cardiomyopathies (HCM) [11–13]. All of these pathological subsets may be characterized by asymmetric hypertrophy of the basal septum, which narrows the left ventricular outflow tract (LVOT) and may contribute to conduction disorders or mechanical complications such as incomplete stent expansion or even distal embolization of THV [13–15].

Although it has been described that isolated TAVI in patients with HCM may lead to postprocedural hemodynamic compromise by unmasking LVOT obstruction or prosthesis migration, there is a discrepancy in the literature regarding the impact of septal hypertrophy on outcomes in TAVI [16].

Understanding the differences in outcomes between TAVI in patients with septal hypertrophy and those with normal IVSD may help in risk stratification, patient counseling, and clinical decision making. Therefore, we herein present a single-center analysis of acute outcomes after TAVI in patients with normal and increased IVSD.

## Methods

### Patients

**Fig. 1:**
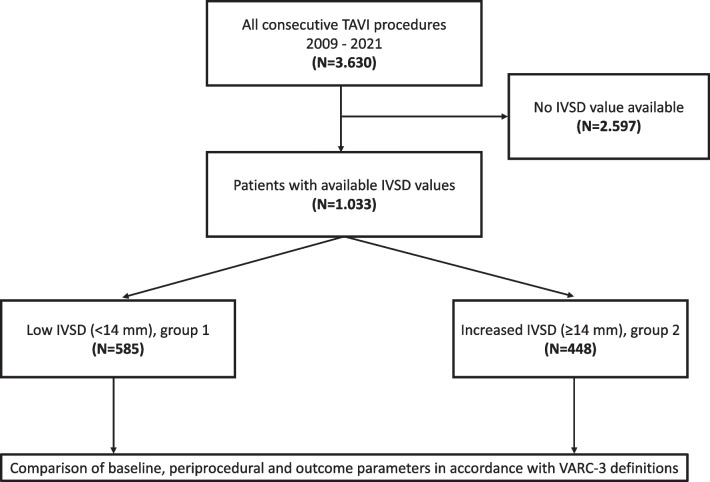
Patient flow during study. IVSD, interventricular septum depth; TAVI, transcatheter aortic valve implantation; VARC-3, valve academic research consortium 3

From 2009 to 2021, a total of 3630 patients received TAVI at our center. Patients with available IVSD measurements by transthoracic (TTE) or transesophageal echocardiography (TEE) were identified and included for analysis. After exclusion of patients that underwent valve-in-valve procedures, 1033 patients remained for analysis. For depiction of patient selection, see Fig. 1.

### Diagnostic work-up

The preprocedural diagnostic work-up followed institutional standards. By routine, all patients received preoperative coronary angiography, TEE, and/or TTE as well as contrast-enhanced, electrocardiogram-gated multi-slice computer tomography (MSCT). Computed tomography data sets were analyzed using the 3mensio Medical Imaging Software (3mensio, Medical Imaging, Bilthoven, Netherlands) for calculation of native aortic annulus dimensions and determination of adequate THV type and size. Additionally, CT scans were used to assess aortic root morphology (e.g., distribution and severity of valvular calcification, aortic root dimensions, and height of coronary ostia take-off), for prediction of the optimal c-arm angulation and for assessment of aorto-iliac vascular status. Postprocedural prosthetic valve function was assessed using TTE.

The IVSD was measured preoperatively by TTE or TEE. Septal hypertrophy was defined as an IVSD ≥ 14 mm according to the definition used in the Framingham Heart Study and was used as cut-off between cohorts [15].

### Data acquisition and statistical analysis

Data acquisition was performed anonymized and retrospectively. Therefore, in accordance with German law, no ethical approval was needed, and informed patient consent was waived. Baseline, periprocedural, and outcome parameters of patients with normal IVSD (< 14 mm; group 1) and increased IVSD (≥ 14 mm; group 2) were compared. Clinical outcomes were adjudicated in accordance with the updated standardized Valve Academic Research Consortium-3 (VARC-3) definitions [17]. Baseline dichotomous and categorical variables were summarized by frequencies and percentages. These were compared between study groups using Fisher’s exact test or Chi-squared test when applicable. Continuous variables were described by mean and standard deviation. They were compared between study groups using the two-sided Student’s *t*-test.

Clinical outcome parameters as well as comparisons of combined VARC-3 endpoint device success and technical success were adjusted for baseline differences between groups using logistic and linear regression analysis. Adjustment factors were gender, aortic valve baseline mean pressure gradient (Pmean), EuroSCORE II, and number of patients with severely reduced ejection fraction (LVEF ≤ 30%). Adjusted odds ratios, 95% confidence intervals, and *p*-values were reported.

The level of significance was set at α = 0.05 for all analyses. Statistical analyses were computed using IBM SPSS version 27.0.0.0 and RStudio 1.3.1093 statistical software.

## Results

### Baseline demographics

Between 2009 and 2021, a total of 1033 patients with available IVSD measurements underwent TAVI at our center. TTE and TEE at baseline confirmed severe AS in all patients. An IVSD < 14 mm (group 1) was detected in 56.6% (*N* = 585), and an IVSD ≥ 14 mm (group 2) was detected in 43.4% (*N* = 448) of patients. In addition, the presence of a functionally relevant systolic anterior motion (SAM) phenomenon or LVOT obstruction was ruled out. Overall, baseline demographics showed a mean age of 80.5 ± 6.7 years, an elevated surgical risk profile (EuroSCORE II 6.3% (± 6.5%)), and a high proportion of patients in New York Heart Association (NYHA) functional class ≥ III (78.2% (*N* = 732)). Patients in group 2 were significantly more often male (group 1, 51.5% (*N* = 300), vs. group 2, 61.5% (*N* = 275), *p* = 0.001), had a higher EuroSCORE II (7.2% (± 7.3%) vs. 4.9% (± 4.6%), *p* < 0.001), and presented less frequently with severely reduced LVEF (14.3% (*N* = 83) vs. 8.7% (*N* = 39), *p* = 0.006). Moreover, Pmean was significantly higher in group 2 (26.1 (± 12.2 mmHg) vs. 33.8 ± (15.2 mmHg), *p* < 0.001). Patients in group 2 continued to exhibit a smaller left ventricular end-diastolic diameter (LVEDD) (50.5 (± 9.6 mm) vs. 47.7 ± (8.7 mm), *p* < 0.001), as well as a larger velocity time integral (VTI) of the LVOT (17.5 (± 5.7 cm) vs. 19.6 ± (6.4 cm), *p* < 0.001).

No significant differences regarding age, rates of extracardiac arthropathy, coronary artery disease, and baseline effective orifice area were found.

A detailed overview of baseline demographics of the study population is given in Table 1.

**Table 1:** Baseline demographics and preprocedural diagnostics

	TAVI IVSD < 14 mm (*N* = 585)	TAVI IVSD ≥ 14 mm (*N* = 448)	Total (*N* = 1033)	*p*-value
Age (years), mean (SD)	80.2 (6.8)	80.9 (6.6)	80.5 (6.7)	0.109
Male gender, *n* (%)	300 (51.5)	275 (61.5)	575 (55.8)	**0.001**
EuroSCORE II, mean (SD)	7.2 (7.3)	4.9 (4.6)	6.3 (6.5)	** < 0.001**
Ejection fraction, *n* (%)				
Normal (> 50%)	305 (52.7)	288 (64.4)	593 (57.7)	** < 0.001**
Mild–moderate (30–50%)	191 (33.0)	120 (26.8)	311 (30.3)	**0.034**
Severe (< 30%)	83 (14.3)	39 (8.7)	122 (11.9)	**0.006**
Extracardiac artheropathy, *n* (%)	145 (24.8)	88 (19.6)	233 (22.6)	0.145
Prior TIA/stroke, *n* (%)	92 (15.9)	64 (14.4)	156 (15.2)	0.814
IVSD (mm), mean (SD)	11.6 (1.4)	15.2 (1.6)	13.2 (2.3)	** < 0.001**
Coronary artery disease, *n* (%)	363 (66.1)	281 (66.8)	644 (66.4)	0.838
NYHA IV, *n* (%)	82 (14.2)	44 (11.1)	126 (13.5)	0.059
NYHA ≥ III, *n* (%)	440 (81.9)	292 (73.2)	732 (78.2)	**0.001**
BMI (kg/m^2^), mean (SD)	26.8 (4.9)	27.8 (9.5)	27.2 (7.3)	0.052
Creatinine (mg/dL), mean (SD)	1.4 (1.0)	1.4 (1.1)	1.4 (1.1)	0.791
Baseline EOA (AV) (cm^2^), mean (SD)	0.8 (0.2)	0.8 (0.7)	0.8 (0.5)	0.099
Pmean (AV) (mmHg), mean (SD)	26.1 (12.2)	33.8 (15.2)	29.6 (14.1)	** < 0.001**
LVEDD (mm), mean (SD)	50.5 (9.6)	47.7 (8.7)	49.3 (9.3)	** < 0.001**
AV-VTI (cm), mean (SD)	73.8 (22.4)	84.3 (23.0)	78.3 (23.3)	** < 0.001**
LVOT-VTI (cm), mean (SD)	17.5 (5.7)	19.6 (6.4)	18.4 (6.1)	** < 0.001**
Aortic annulus area (mm^2^), mean (SD)	467.2 (87.8)	480.4 (82.9)	472.5 (86.1)	0.072

*AV* aortic valve, *BMI* body mass index, *EOA* effective orifice area, *IVSD* interventricular septum depth, *LVEDD* left ventricular end-diastolic diameter, *LVOT* left ventricular outflow tract, *NYHA* New York Heart Association, *SD* standard deviation, *STJ* sinutubular junction, *TAVI* transcatheter aortic valve implantation, *TIA* transient ischemic attack, *VTI* velocity time integral

**Fig. 2:**
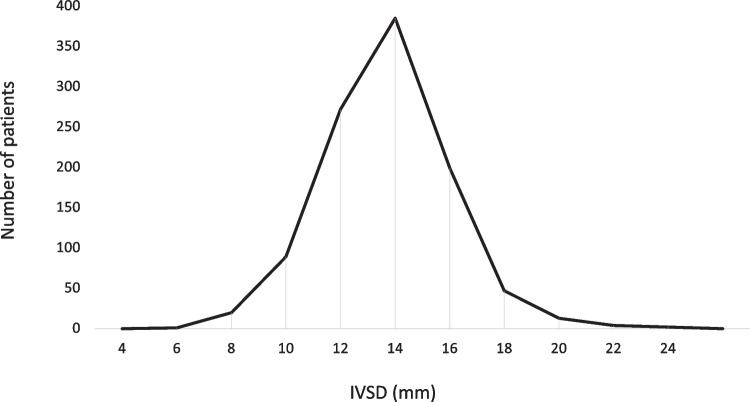
Distribution of interventricular septum depth. IVSD, interventricular septum depth

Distribution of IVSD is shown in Fig. 2.

### Periprocedural parameters

Transfemoral access was utilized in 84.1% (*N* = 859) of the entire study cohort without significant differences between groups. Balloon-expandable THV were most frequently used across groups without significant differences in frequency (*p* = 0.208). Rates of balloon postdilatation showed no significant differences.

Prosthesis label size was significantly higher in patients with relevant septal hypertrophy (26.6 mm (± 2.6 mm) vs. 27.2 mm (± 3.3 mm), *p* = 0.007). In the entire study cohort, low rates of valve malpositioning (1.6% (*N* = 17)) or conversion to cardiopulmonary bypass (CPB) (0.7% (*N* = 7)) were observed with no significant differences between groups.

A detailed overview of periprocedural parameters is given in Table 2.

**Table 2:** Periprocedural outcome parameters

	TAVI IVSD < 14 mm (*N* = 585)	TAVI IVSD ≥ 14 mm (*N* = 448)	Total (*N* = 1033)	*p*-value
Transfemoral access,* n* (%)	478 (82.6)	381 (86.0)	859 (83.2)	0.157
Prosthesis, *n* (%)				
Edwards Sapien (XT/3/3 Ultra)	237 (40.8)	199 (44.8)	436 (42.5)	0.208
Medtronic CoreValve (Evolut R/Pro/PRO +)	161 (27.7)	149 (33.6)	310 (30.2)	**0.046**
Boston Acurate (TA/neo-TF)	106 (18.2)	58 (13.1)	164 (16.0)	**0.024**
Abbott Portico/Navitor	31 (5.3)	15 (3.4)	46 (4.9)	0.132
JenaValve	23 (4.0)	11 (2.5)	34 (3.3)	0.188
Boston Lotus	8 (1.4)	4 (0.9)	12 (1.2)	0.480
Other	15 (2.6)	8 (1.8)	23 (2.2)	0.401
Prosthesis label size (mm), mean (SD)	26.7 (2.6)	27.2 (3.3)	26.9 (2.8)	**0.007**
Postdilatation, *n* (%)	186 (32.7)	164 (37.7)	350 (34.9)	0.264
Predilatation, *n* (%)	401 (70.4)	342 (77.7)	743 (73.6)	**0.022**
Valve malpositioning, *n* (%)	8 (1.4)	9 (2.0)	17 (1.6)	0.363
Conversion to CPB, *n* (%)	4 (0.7)	3 (0.2)	7 (0.7)	0.545
Length of ICU stay (days), mean (SD)	2.3 (4.6)	1.8 (3.0)	2.1 (4.0)	0.276
Length of hospital stay (days), mean (SD)	*9.7 (8.3)*	*8.2 (5.7)*	*9.0 (7.3)*	0.188

*CPB* cardiopulmonary bypass, *ICU* intensive care unit, *IVSD* interventricular septum depth, *SD* standard deviation, *TAVI* transcatheter aortic valve implantation

### Echocardiographic and clinical outcome parameters

Resultant Pmean of the entire study population as assessed by TTE prior to discharge decreased from 29.6 mmHg ± 14.1 mmHg to 8.3 mmHg ± 4.5 mmHg (*p* < 0.01). However, patients in group 2 showed a higher postprocedural Pmean (7.8 mmHg (± 4.1 mmHg) vs. 8.9 mmHg (± 4.5 mmHg), *p* = 0.046) compared to patients in group 1. Moreover, patients in group 2 showed a trend towards higher rates of Pmean ≥ 20 mmHg (1.9% (*N* = 10) vs. 4.3% (*N* = 18), *p* = 0.093).

Furthermore, postoperative echocardiography revealed PVL ≥ moderate in 2.9% (*N* = 23) of patients with no significant differences between groups.

There were no differences regarding length of ICU stay, rates of major vascular complications, bleeding, or frequency of acute kidney injury. Disabling stroke occurred in 2.5% (*N* = 13%) of patients in group 1 and in 2.8% (*N* = 10%) of patients in group 2 (*p* = 0.217).

In total, 30-day mortality was 5.1% (*N* = 30) in group 1 and 4.5% (*N* = 20) in group 2 (*p* = 0.758). Even after a 1-year follow-up, there was no statistically significant difference in mortality (23.8% (*N* = 139) vs. 17.4% (*N* = 78), *p* = 0.409)) between the groups.

Comparison of VARC-3 composite endpoint technical success and device success was adjusted for baseline differences between the groups using logistic regression analysis.

**Fig. 3:**
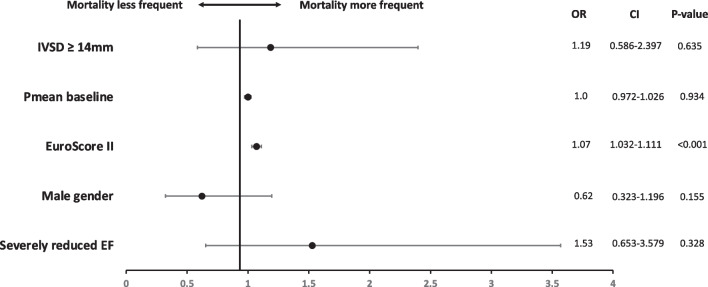
Final model of multivariate logistic regression for 30-day survival. CI, confidence interval; EF, ejection fraction; IVSD, interventricular septum depth; OR, odds ratio

Increased IVSD was not associated with lower rates of technical success (92.6% (*N* = 542) vs. 92.6% (*N* = 415), adjusted OR = 0.870, 95% CI 0.487–1.556, *p* = 0.639) and device success (90.0% (*N* = 522) vs. 87.6% (*N* = 388), adjusted OR = 1.167, 95% CI 0.715–1.905, *p* = 0.538) compared to patients with normal IVSD (see Fig. 3). When excluding non-transfemoral TAVI cases, statistical analyses presented similar results (see Supplementary Table 1–3)*.* Even with a higher IVSD cut-off ≥ 16 mm, no significant differences were observed in the clinical outcome parameters between the groups. However, an IVSD ≥ 16 mm was significantly associated with an increased number of patients exhibiting a postprocedural Pmean ≥ 20 mmHg (see Supplementary Table 4–6).

A detailed overview of echocardiographic and detailed clinical outcome parameters is summarized in Table 3.

**Table 3:** Echocardiographic and clinical outcome parameters

	TAVI IVSD < 14 mm (*N* = 585)	TAVI IVSD ≥ 14 mm (*N* = 448)	Total (*N* = 1033)	Adjusted *p*-value*
Pacemaker implantation, *n* (%)	77 (13.4)	61 (13.8)	138 (13.6)	0.788
Mean gradient (mmHg), mean (SD)	7.8 (4.1)	8.9 (4.9)	8.3 (4.5)	**0.046**
Mean gradient ≥ 20 mmHg, *n* (%)	10 (1.9)	18 (4.3)	28 (3.0)	0.093
PVL ≥ mild*, n* (%)	161 (35.2)	111 (26.6)	272 (28.3)	0.977
PVL ≥ moderate, *n* (%)	14 (3.1)	9 (2.6)	23 (2.9)	0.993
Major vascular complication, *n* (%)	29 (5.0)	25 (5.7)	54 (5.3)	0.866
Bleeding ≥ BARC type III, *n* (%)	22 (3.8)	22 (5.0)	44 (4.3)	0.711
AKIN grade ≥ II, *n* (%)	35 (6.1)	11 (2.5)	46 (4.5)	0.082
Non-disabling stroke, *n* (%)	10 (1.9)	4 (1.1)	14 (1.6)	0.687
Disabling stroke, *n* (%)	13 (2.5)	10 (2.8)	23 (2.6)	0.217
Myocardial infarction, *n* (%)	3 (0.5)	3 (0.7)	6 (0.6)	0.851
30-day mortality, *n* (%)	30 (5.1)	20 (4.5)	50 (4.8)	0.758
VARC-III device success, *n* (%)	522 (90.0)	388 (87.6)	910 (88.9)	0.538
VARC-III technical success, *n* (%)	542 (92.6)	415 (92.6)	957 (92.6)	0.639
1-year mortality, *n* (%)	*139 (23.8)*	*78 (17.4)*	*217 (21.0)*	0.409

*AKIN* acute kidney injury, *CPB* cardiopulmonary bypass, *PVL* paravalvular leakage, *SD* standard deviation, *TAVI* transcatheter aortic valve implantation, *TIA* transient ischemic attack

^*^Adjusted for gender, aortic valve baseline mean pressure gradient (Pmean), EuroSCORE II, and number of patients with severely reduced ejection fraction (LVEF ≤ 30%)

## Discussion

The main findings of our retrospective single-center analysis are (I) TAVI in patients with significant septal hypertrophy is technical feasible and presents a reasonable safety and efficacy profile, (II) increased postprocedural Pmean in patients with significant hypertrophy did not impact rates of VARC-3-adjudicated composite endpoint device success and technical success, and (III) moreover, septal hypertrophy was not associated with higher rates of relevant PVL, PPM, or 30-day mortality.

A severe stenosis of the aortic valve often leads to a concentric hypertrophy of the left ventricular myocardium as a result of increased afterload. Besides a global concentric hypertrophy, a asymmetric thickening of the basal septum can also be an adaptive mechanism [18, 19].

A significant septal hypertrophy as a risk factor for adverse clinical outcome in TAVI procedures has been described [16, 20]. However, inconsistent results of previous reports hamper clear conclusions for treatment of this special subset of patients.

Bandyopadhyay and co-workers reported that the presence of septal hypertrophy in patients undergoing TAVI is associated with increased in-hospital mortality and increased rates of acute kidney injury as well as postprocedural cardiogenic shock [16]. The authors interpret these results as a consequence of acute afterload reduction after TAVI with consecutive increase of blood flow in the LVOT and unmasking of a relevant LVOT obstruction by formation of a SAM phenomenon [21]. Further explanations of impaired outcomes include a possible incomplete THV stent expansion of the ventricular stent part with consecutive flow acceleration caused by the thickening of the septum. This assumption is supported by a retrospective analysis from Kiefer and co-workers including 296 patients undergoing TAVI. Here, an increased rate of postdilatation in patients with septal hypertrophy was seen, but septal hypertrophy was not associated with adverse mechanical or conduction complications at 30 days [11]. In our 12-year single-center experience, we were able to confirm these results with a significantly larger number of patients. It should be emphasized that also in our analysis, the rate of PPM was not affected by the presence of septal hypertrophy, although there is an increased anatomical proximity to the bundle of His due to a thicker septum. Systematic high implantation of next-generation advanced THV systems over time may have contributed to this finding [22, 23]. However, since there were no significant differences in PPM between the groups over the 12-year period of enrolment, it can be inferred that the observed effect was independent of the presence of septal hypertrophy. Whether the postprocedural increased transvalvular aortic valve gradient in our study was caused by incomplete stent expansion of the THV or by the residual subvalvular stenotic component could not be determined. However, the herein seen increased postinterventional transvalvular pressure gradients in patients with significant septal hypertrophy are particularly notable because of a higher proportion of utilized supra-annular THV in this group, which are traditionally considered to provide better hemodynamics compared to intra-annular THV.

The present study is the largest report on TAVI in patients with significant septal hypertrophy. Herein, we show feasibility of TAVI in patients with septal hypertrophy ≥ 14 mm with low procedural event rates and a reasonable acute efficacy within the first 30 days. Septal hypertrophy was defined according to the Framingham Heart Study [11, 15]. However, there is still no clear consensus on the definition of septal hypertrophy, leading to heterogeneous results which are difficult to compare. A higher cut-off value for the thickness of the septal wall may lead to different results. We and others suggest that all patients diagnosed with AS should be screened preprocedurally for septal hypertrophy. Septal thickening ≥ 14 mm should be addressed especially in heart team discussion to provide patient-specific treatment decisions including potential bailout strategies. For example, pre-TAVI alcohol septal ablation to reduce septal thickness improves valve deployment and procedural outcomes [24, 25]. In cases of preprocedural LVOT obstruction including SAM, surgical septal myectomy with subsequent prosthesis implantation may be taken into consideration in patients with acceptable risk for surgery [26].

In summary, the results of this study suggest that TAVI in patients with severe AS and concomitant hypertrophy of the ventricular septum represents a feasible procedure with high procedural safety and acute efficacy.

Nevertheless, a heart team discussion remains mandatory for patients with relevant septal hypertrophy.

### Limitations

The main limitations of our study are its retrospective and non-randomized single-center design.

Baseline characteristics of the groups significantly differed with regard to gender, EuroSCORE II, rates of severely reduced LVEF, and aortic valve Pmean. This issue was at least partially addressed by multiple adjustments for confounding imbalances in the comparisons of clinical outcome parameters. Due to the retrospective design of the study, any drawn conclusions can only serve to generate hypotheses.

## Conclusion

In this study, TAVI in the presence of septal hypertrophy was performed with technical feasibility, procedural safety, and acute efficacy in the majority of patients. Although significant, postprocedural aortic valve Pmean is not excessively elevated in patients with increased septal hypertrophy. This results suggest that these echocardiographic finding plays no crucial role in the acute postoperative course. However, how these differences in hemodynamic findings may accelerate during mid- and long-term outcomes which needs to be evaluated in further investigations.

### Impact on daily practice

TAVI in the presence of septal hypertrophy is associated with technical feasibility, procedural safety, and acute efficacy in the majority of patients and should be performed when indicated according to guidelines. Nevertheless, a heart team discussion remains mandatory for patients with relevant septal hypertrophy.

## Supplementary Information

ESM 1 (DOCX 36.8 kb)

ESM 2 (DOCX 31.2 kb)

## Data Availability

Due to German data protection laws, data sharing will only be possible after approval by the ethics committee of the medical chamber Hamburg. For data request, please contact the corresponding author.

## Abbreviations

AS: Aortic stenosis
BMI: Body mass index
CBP: Cardiopulmonary bypass
EOA: Effective orifice area
HCM: Hypertrophic cardiomyopathy
MSCT: Multi-slice computer tomography
NYHA: New York Heart Association
ICU: Intensive care unit
IVSD: Interventricular septum depth
LVEDD: Left ventricular end-diastolic diameter
LVEF: Left ventricular ejection fraction
LVOT: Left ventricular outflow tract
Pmean: Mean pressure gradient
PPM: Permanent pacemaker implantation
PVL: Paravalvular leakage
SAM: Systolic anterior motion
SD: Standard deviation
TAVI: Transcatheter aortic valve implantation
TEE: Transesophageal echocardiography
THV: Transcatheter heart valve
TIA: Transient ischemic attack
TTE: Transthoracic echocardiography
VARC-3: Valve Academic Research Criteria-3
VTI: Velocity time integral
