# Supplementary material for: Transcatheter aortic valve implantation in patients with significant septal hypertrophy
Source: Clin Res Cardiol. 2024 Mar 11;114(3):332–40. doi: 10.1007/s00392-024-02432-3 (PMC11914327; doi:10.1007/s00392-024-02432-3)
Supplement: Supplementary file 1 — (DOCX 36.8 kb) [file 392_2024_2432_MOESM1_ESM.docx]

**Supplementary Table 1: Baseline demographics and preprocedural diagnostics**

|  | **tfTAVI**  **IVSD < 14mm**  (N=478) | **tfTAVI**  **IVSD ≥ 14mm**  (N=381) | **Total**  (N=859) | **p-value** |
| --- | --- | --- | --- | --- |
| **Age (years), mean (SD)** | 80 (6.8) | 81 (6.7) | 81 (6.8) | 0.065 |
| **Male Gender, n (%)** | 235 (49.3) | 231 (60.9) | 466 (54.4) | **<0.001** |
| **EuroSCORE II, mean (SD)** | 6.8 (7.4) | 4.3 (4.2) | 5.8 (6.4) | **<0.001** |
| **Ejection fraction, n (%)** |  |  |  |  |
| Normal (>50%) | 247 (52.3) | 244 (64.2) | 491 (57.6) | **<0.001** |
| Mild-Moderate (30-50%) | 151 (32.0) | 102 (26.8) | 253 (29.7) | 0.102 |
| Severe (<30%) | 74 (15.7) | 34 (8.9) | 108 (12.7) | **0.003** |
| **Extracardiac Artheropathy, n (%)** | 91 (19.0) | 59 (15.5) | 150 (17.5) | 0.385 |
| **Prior TIA / Stroke, n (%)** | 70 (14.6) | 50 (13.1) | 120 (14.0) | 0.780 |
| **IVSD (mm), mean (SD)** | 11.6 (1.4) | 15.2 (1.6) | 13.2 (2.3) | **<0.001** |
| **Coronary artery disease, n (%)** | 282 (63.7) | 230 (64.8) | 512 (64.1) | 0.740 |
| **NYHA IV, n (%)** | 64 (15.0) | 38 (11.4) | 102 (13.4) | 0.159 |
| **NYHA ≥ III, n (%)** | 344 (80.4) | 235 (70.1) | 579 (76.2) | **0.002** |
| **BMI (kg/m^2^), mean (SD)** | 26.8 (4.8) | 27.9 (10.1) | 27.3 (7.6) | 0.052 |
| **Creatinine (mg/dL), mean (SD)** | 1.4 (1.0) | 1.4 (1.1) | 1.4 (1.0) | 0.937 |
| **Baseline EOA (cm²) (AV), mean (SD)** | 0.8 (0.2) | 0.8 (0.8) | 0.8 (0.5) | 0.075 |
| **Mean gradient (mmHg), mean (SD)** | 26.5 (12.7) | 35.2 (15.5) | 30.4 (14.7) | **<0.001** |
| **LVEDD (mm), mean (SD)** | 50.0 (9.7) | 47.3 (8.8) | 48.8 (9.4) | **<0.001** |
| **AV-VTI (cm), mean (SD)** | 74.0 (23.0) | 86.2 (23.3) | 79.4 (23.9) | **<0.001** |
| **LVOT-VTI (cm), mean (SD)** | 17.7 (6.0) | 20.0 (6.6) | 18.7 (6.4) | **<0.001** |
| **Aortic annulus area (mm^2^), mean (SD)** | 465.8 (88.6) | 482.3 (85.0) | 472.4 (87.5) | **0.044** |
|  | **Supplementary Table 1: Baseline demographics.** AV Aortic valve, *BMI Body mass index,*  *EOA Effective orifice area, IVSD Interventricular septum depth, LVEDD Left ventricular end-diastolic diameter, LVOT Left ventricular outflow tract, NYHA New York Heart Association,*  *SD Standard deviation, STJ Sinutubular junction, TAVI Transcatheter aortic valve implantation,*  *tf Transfemoral, TIA Transient ischemic attack, VTI Velocity time integral.* |  |  |  |

**Supplementary Table 2: Periprocedural outcome parameters**

|  | **tfTAVI**  **IVSD < 14 mm**  (N=478) | **tfTAVI**  **IVSD ≥ 14 mm**  (N=381) | **Total**  (N=859) | **p-value** |
| --- | --- | --- | --- | --- |
| **Prosthesis, n (%)** |  |  |  |  |
| Edwards Sapien (XT/3/3 Ultra) | 194 (40.6) | 170 (44.6) | 364 (42.4) | 0.235 |
| Medtronic CoreValve  (Evolut R/Pro/PRO+) | 155 (32.4) | 145 (38.1) | 300 (34.9) | 0.085 |
| Boston Acurate (TA/neo-TF) | 81 (16.9) | 46 (12.1) | 127 (14.8) | **0.046** |
| Abbott Portico/Navitor | 30 (6.3) | 14 (3.7) | 44 (5.1) | 0.086 |
| JenaValve | 1 (0.21) | 0 (0.0) | 1 (0.1) | 0.372 |
| Boston Lotus | 7 (1.5) | 4 (1.0) | 11 (1.3) | 0.591 |
| Other | 10 (2.1) | 2 (0.5) | 12 (1.4) | 0.052 |
| **Prosthesis label size (mm), mean (SD)** | 26.8 (2.7) | 27.4 (3.2) | 27.1 (2.9) | **0.002** |
| **Postdilatation, n (%)** | 163 (34.1) | 145 (38.1) | 308 (35.9) | 0.476 |
| **Predilatation, n (%)** | 324 (67.8) | 295 (77.4) | 619 (72.1) | **0.006** |
| **Valve malpositioning, n (%)** | 6 (1.3) | 6 (1.6) | 12 (1.4) | 0.692 |
| **Conversion to CPB, n (%)** | 4 (0.8) | 2 (0.5) | 6 (0.7) | 0.586 |
| **Length of ICU stay (days), mean (SD)** | 2.2 (4.6) | 1.8 (3.2) | 2.0 (4.1) | 0.143 |
| **Length of hospital stay (days), mean (SD)** | 9.5 (8.6) | 7.9 (5.6) | 8.8 (7.5) | **0.002** |
|  |  |  |  |  |

**Supplementary Table 2: Periprocedural outcome parameters.** *CPB Cardiopulmonary bypass, ICU Intensive care unit, IVSD Interventricular septum depth, SD Standard deviation,*

*TAVI Transcatheter aortic valve implantation, tf Transfemoral.*

**Supplementary Table 3: Echocardiographic and clinical outcome parameters**

**Table 3: Echocardiographic and clinical outcome parameters.** *AKIN: Acute kidney injury.*

*CPB Cardiopulmonary bypass, PVL Paravalvular leakage, SD Standard deviation, TAVI Transcatheter aortic valve implantation, tf Transfemoral, TIA Transient ischemic attack.*

***** Adjusted for gender, aortic valve baseline mean pressure gradient (Pmean), EuroSCORE II and number of patients with severely reduced ejection fraction (LVEF ≤ 30%)

|  | **tfTAVI**  **IVSD < 14 mm**  (N=478) | **tfTAVI**  **IVSD ≥ 14 mm**  (N=381) | **Total**  (N=859) | **Adjusted**  **p-value*** |
| --- | --- | --- | --- | --- |
| **Pacemaker implantation, n (%)** | 54 (11.3) | 48 (12.7) | 102 (12.0) | 0.872 |
| **Mean gradient (mmHg), mean (SD)** | 7.7 (4.2) | 8.8 (4.8) | 8.2 (4.5) | **<0.001** |
| **Mean gradient ≥20mmHg, n (%)** | 9 (1.9) | 14 (3.7) | 23 (2.7) | 0.602 |
| **PVL ≥ mild, n (%)** | 125 (28.0) | 84 (23.3) | 209 (25.9) | 0.432 |
| **PVL ≥ moderate, n (%)** | 14 (3.1) | 6 (0.3) | 20 (2.5) | 0.353 |
| **Major vascular complication, n (%)** | 22 (4.6) | 21 (5.8) | 43 (5.0) | 0.469 |
| **Bleeding ≥ BARC type III, n (%)** | 13 (2.8) | 17 (4.5) | 30 (3.5) | 0.466 |
| **AKIN ≥ II, n (%)** | 24 (5.0) | 8 (2.1) | 32 (3.8) | 0.373 |
| **Stroke, n (%)** | 19 (4.5) | 11 (3.7) | 30 (4.2) | 0.315 |
| **Non-disabling stroke, n (%)** | 8 (1.9) | 4 (1.3) | 12 (1.7) | 0.676 |
| **Disabling stroke, n (%)** | 11 (2.6) | 7 (2.3) | 18 (2.5) | 0.346 |
| **Myocardial infarction, n (%)** | 1 (0.2) | 2 (0.5) | 3 (0.4) | 0.760 |
| **30-day mortality, n (%)** | 23 (4.8) | 12 (3.1) | 35 (4.1) | 0.829 |
| **VARC-III device success, n (%)** | 423 (89.2) | 332 (87.8) | 755 (88.6) | 0.928 |
| **VARC-III technical success, n (%)** | 438 (91.6) | 353 (92.7) | 790 (92.0) | 0.602 |
| **1-year mortality, n (%)** | 103 (21.5) | 51 (13.4) | 154 (17.9) | 0.064 |
|  |  |  |  |  |
