# Supplementary material for: Transcatheter aortic valve implantation in patients with significant septal hypertrophy
Source: Clin Res Cardiol. 2024 Mar 11;114(3):332–40. doi: 10.1007/s00392-024-02432-3 (PMC11914327; doi:10.1007/s00392-024-02432-3)
Supplement: Supplementary file 2 — (DOCX 31.2 kb) [file 392_2024_2432_MOESM2_ESM.docx]

**Supplementary Table 4: Baseline demographics and preprocedural diagnostics**

|  | **TAVI**  **IVSD < 16mm**  (N=906) | **TAVI**  **IVSD ≥ 16mm**  (N=127) | **Total**  (N=1033) | **p-value** |
| --- | --- | --- | --- | --- |
| **Age (years), mean (SD)** | 80.5 (6.6) | 80.9 (7.2) | 80.5 (6.7) | 0.541 |
| **Male Gender, n (%)** | 494 (54.5) | 79 (62.0) | 575 (55.8) | 0.236 |
| **EuroSCORE II, mean (SD)** | 6.5 (6.7) | 4.7 (4.4) | 6.3 (6.5) | **0.002** |
| **Ejection fraction, n (%)** |  |  |  |  |
| Severe (<30%) | 114 (12.7) | 8 (6.3) | 122 (11.9) | **0.038** |
| **Prior TIA / Stroke, n (%)** | 132 (14.6) | 24 (18.9) | 156 (15.2) | 0.442 |
| **IVSD (mm), mean (SD)** | 12.6 (1.8) | 17.1 (1.7) | 13.2 (2.3) | **<0.001** |
| **NYHA ≥ III, n (%)** | 656 (79.4) | 76 (69.1) | 732 (78.2) | **0.014** |
| **BMI (kg/m^2^), mean (SD)** | 27.3 (7.6) | 27.0 (5.1) | 27.2 (7.3) | 0.700 |
| **Baseline EOA (AV) (cm²), mean (SD)** | 0.8 (0.5) | 0.7 (0.2) | 0.8 (0.5) | 0.111 |
| **Pmean (AV) (mmHg), mean (SD)** | 28.4 (13.4) | 37.3 (15.4) | 29.6 (14.1) | **<0.001** |
| **LVEDD (mm), mean (SD)** | 49.8 (9.2) | 45.8 (9.7) | 49.3 (9.3) | **<0.001** |
| **AV-VTI (mm), mean (SD)** | 76.9 (22.7) | 88.5 (24.7) | 78.3 (23.3) | **<0.001** |
| **LVOT-VTI (cm), mean (SD)** | 18.2 (6.0) | 20.0 (6.6) | 18.4 (6.1) | **0.002** |
| **Aortic annulus area (mm^2^), mean (SD)** | 472.0 (87.5) | 475.7 (75.8) | 472.5 (86.1) | 0.740 |

**Supplementary Table 4: Baseline demographics.** AV Aortic valve, *BMI Body mass index,*

*EOA Effective orifice area, IVSD Interventricular septum depth, LVEDD Left ventricular end-diastolic diameter, LVOT Left ventricular outflow tract, NYHA New York Heart Association, SD Standard deviation, STJ Sinutubular junction, TAVI Transcatheter aortic valve implantation, TIA Transient ischemic attack, VTI Velocity time integral.*

**Supplementary Table 5: Periprocedural outcome parameters**

|  | **TAVI**  **IVSD < 16 mm**  (N=906) | **TAVI**  **IVSD ≥ 16 mm**  (N=127) | **Total**  (N=1033) | **p-value** |
| --- | --- | --- | --- | --- |
| **Transfemoral access, n (%)** | 748 (82.6) | 111 (87.4) | 859 (84.1) | 0.172 |
| **Prosthesis label size (mm), mean (SD)** | 26.9 (2.9) | 27.3 (3.0) | 26.9 (2.8) | 0.117 |
| **Conversion to CPB, n (%)** | 5 (0.6) | 2 (1.6) | 7 (0.7) | 0.188 |
| **Length of ICU stay (days), mean (SD)** | 2.2 (4.3) | 1.6 (1.4) | 2.1 (4.0) | 0.003 |
| **Length of hospital stay (days), mean (SD)** | 9.1 (7.3) | 8.6 (7.2) | 9.0 (7.3) | 0.526 |
| **Supplementary Table 5: Periprocedural outcome parameters.** *CPB Cardiopulmonary bypass, ICU Intensive care unit, IVSD Interventricular septum depth, SD Standard deviation, TAVI Transcatheter aortic valve implantation.* |  |  |  |  |

**Supplementary Table 6: Echocardiographic and clinical outcome parameters**

|  | **TAVI**  **IVSD < 16 mm**  (N=906) | **TAVI**  **IVSD ≥ 16 mm**  (N=127) | | **Total**  (N=1033) | **Adjusted**  **p-value*** |
| --- | --- | --- | --- | --- | --- |
| **Pacemaker implantation, n (%)** | 120 (13.5) | | 18 (14.3) | 138 (13.6) | 0.314 |
| **Mean gradient ≥20mmHg, n (%)** | 19 (2.3) | | 9 (7.5) | 28 (3.0) | **0.018** |
| **PVL ≥ mild, n (%)** | 236 (28.0) | | 36 (30.0) | 272 (28.3) | 0.056 |
| **Major vascular complication, n (%)** | 46 (5.1) | | 8 (6.3) | 54 (5.3) | 0.702 |
| **Bleeding ≥ BARC type III, n (%)** | 39 (4.4) | | 5 (4.0) | 44 (4.3) | 0.848 |
| **AKIN Grade ≥ II, n (%)** | 43 (4.8) | | 3 (2.4) | 46 (4.5) | 0.950 |
| **Non-disabling stroke, n (%)** | 13 (1.7) | | 1 (0.9) | 14 (1.6) | 0.813 |
| **Disabling stroke, n (%)** | 21 (2.7) | | 2 (1.9) | 23 (2.6) | 0.802 |
| **Myocardial infarction, n (%)** | 5 (0.6) | | 1 (0.8) | 6 (0.6) | 0.997 |
| **30-day mortality, n (%)** | 47 (5.2) | | 3 (2.4) | 50 (4.8) | 0.696 |
| **VARC-III device success, n (%)** | 797 (88.8) | | 113 (90.0) | 910 (88.9) | 0.289 |
| **VARC-III technical success, n (%)** | 840 (92.7) | | 117 (92.1) | 957 (92.6) | 0.322 |
| **1-year mortality, n (%)** | 199 (22.0) | | 18 (14.2) | 217 (21.0) | 0.286 |
| **Supplementary Table 6: Echocardiographic and clinical outcome parameters.** *AKIN Acute kidney injury,* *CPB Cardiopulmonary bypass, PVL Paravalvular leakage, SD Standard deviation,*  *TAVI Transcatheter aortic valve implantation, TIA Transient ischemic attack.*  ***** Adjusted for aortic valve baseline mean pressure gradient (Pmean), EuroSCORE II and number of patients with severely reduced ejection fraction (LVEF ≤ 30%). |  | |  |  |  |
